# Salmonella Induced IL-23 and IL-1β Allow for IL-12 Production by Monocytes and Mϕ1 through Induction of IFN-γ in CD56^+^ NK/NK-Like T Cells

**DOI:** 10.1371/journal.pone.0008396

**Published:** 2009-12-21

**Authors:** Diederik van de Wetering, Roelof A. de Paus, Jaap T. van Dissel, Esther van de Vosse

**Affiliations:** Department of Infectious Diseases, Leiden University Medical Center, Leiden, The Netherlands; Centre de Recherche Public de la Santé (CRP-Santé), Luxembourg

## Abstract

**Background:**

The type-1 cytokine pathway plays a pivotal role in immunity against intracellular bacterial pathogens such as *Salmonellae* and *Mycobacteria*. Bacterial stimulation of pattern recognition receptors on monocytes, macrophages and dendritic cells initiates this pathway, and results in the production of cytokines that activate lymphocytes to produce interferon (IFN)-γ. Interleukin (IL)-12 and IL-23 are thought to be the key cytokines required for initiating a type-1 cytokine immune response to *Mycobacteria* and S*almonellae*. The relative contribution of IL-23 and IL-12 to this process is uncertain.

**Methodology/Principal Findings:**

We show that various TLR agonists induce the production of IL-23 but not IL-12 in freshly isolated human monocytes and cultured human macrophages. In addition, type 1 pro-inflammatory macrophages (Mϕ1) differentiated in the presence of GM-CSF and infected with live *Salmonella* produce IL-23, IL-1β and IL-18, but not IL-12. Supernatants of *Salmonella*-infected Mϕ1 contained more IL-18 and IL-1β as compared with supernatants of Mϕ1 stimulated with isolated TLR agonists, and induced IFN-γ production in human CD56^+^ cells in an IL-23 and IL-1β-dependent but IL-12-independent manner. In addition, IL-23 together with IL-18 or IL-1β led to the production of GM-CSF in CD56^+^ cells. Both IFN-γ and GM-CSF enhanced IL-23 production by monocytes in response to TLR agonists, as well as induced IL-12 production.

**Conclusions/Significance:**

The findings implicate a positive feedback loop in which IL-23 can enhance its release via induction of IFN-γ and GM-CSF. The IL-23 induced cytokines allow for the subsequent production of IL-12 and amplify the IFN-γ production in the type-1 cytokine pathway.

## Introduction

Immunity against intracellular bacterial pathogens such as *Salmonellae* and *Mycobacteria* depends on the type-1 cytokine pathway [1]. This pathway is initiated by bacterial stimulation of pattern recognition receptors on monocytes and macrophages, resulting in the production of cytokines that activate lymphocytes and induce IFN-γ production. The IFN-γ in turn activates monocytes and macrophages, to enhance bactericidal effector mechanisms and to further pro-inflammatory cytokine production. Thus, the type-1 cytokine pathway critically depends on the cross-talk between monocytes/macrophages and lymphocytes. Abdi *et al.* state that IL-12p70 cannot be the primary trigger that initiates Th1 T-cell responses, as it is not produced in response to bacterial stimulation when costimulation in the form of activated T cells or IFN-γ are absent [2]. In addition, in whole blood assays with blood obtained from patients with complete IFN-γR deficiency, no IL-12p70 production can be detected in response to M. *bovis* BCG infection *in vitro* [3]. How the type-1 pathway is initiated, therefore, has remained uncertain.

Interleukin-23 (IL-23) is a cytokine which is produced early in the immune response [4]. Monocytes as well as type 1 macrophages (Mϕ1) produce IL-23 in response to the binding of pathogens and pathogen-associated molecular patterns (PAMPs), such as lipopolysaccharide (LPS), to Toll-like receptors (TLRs) [5], [6]. In contrast to IL-23, for the production of IL-12 in response to PAMPs an additional stimulus such as IFN-γ is required [5]. IL-23 is known to induce IFN-γ production in naïve T cells, in memory T cells [7] and in NK-like T cells [8], thereby potentially providing the necessary, additional stimulus to induce IL-12 production. Next, IL-12 and IL-18 enhance IFN-γ production in NK, NK-like T cells and Th1 cells by binding to their respective receptors [9]. Though IFN-γ is not required for the induction of IL-23, the precise role of IFN-γ in the regulation of IL-23 is not well established.

Granulocyte-Macrophage Colony Stimulating Factor (GM-CSF) is a cytokine produced by NK cells and T cells in response to a variety of stimuli, for instance IL-15 and IL-18 [10]. GM-CSF activates monocytes and enhances their bactericidal activity [11]–[13]. Moreover, monocytes pre-stimulated with GM-CSF secrete increased quantities of tumor necrosis factor (TNF) and IL-1β when stimulated with LPS [14], [15]. Furthermore, GM-CSF induces differentiation of human monocytes into Mϕ1, a macrophage type that is capable of producing IL-23 [5].

In this study we addressed the roles of the cytokines IL-23, IL-1β, IL-18, GM-CSF and IFN-γ in the crosstalk between NK/NK-like T cells and monocytes/macrophages in the early activation of the type-1 cytokine pathway. To this end we determined which TLR agonists induce IL-23 production in human monocytes and macrophages and assessed the roles of GM-CSF and IFN-γ in the regulation of IL-23 production by human monocytes in response to TLR agonists. Furthermore, we explored the role of *Salmonella*-induced IL-23, IL-1β and IL-18 in the induction of IFN-γ in primary human NK/NK-like T cells, and tested the capacities of IL-23, IL-1β and IL-18 to induce GM-CSF and IFN-γ in human NK and NK-like T cells.

## Materials and Methods

### Cells and Culture Conditions

Human CD14^+^ cells and CD56^+^ cells were isolated from buffy coats from healthy donors (Sanquin) by Ficoll-Amidotrizoate density gradient centrifugation and subsequent selection with anti-CD14 MACS beads or anti-CD56 MACS beads (Miltenyi Biotech).

CD14^+^ cells were cultured in RPMI-1630 medium, supplemented with 20 mM GlutaMAX (Gibco/Invitrogen), 10% FCS, 100 U/ml Penicillin, 100 µg/ml Streptomycin (Gibco/Invitrogen). CD56^+^ cells were cultured in Iscove's modified Dulbecco's medium (IMDM, Bio-Whittaker) supplemented with 20 mM GlutaMAX, 10% FCS, 100 U/ml Penicillin, 100 µg/ml Streptomycin. To generate Mϕ1, CD14^+^ cells were cultured for 6 days in RPMI 1640 with 10% FCS and 5 ng/ml GM-CSF (Biosource).

### Cytokine Induction and Measurement

To determine cytokine production by monocytes, CD14^+^ beads isolated cells were seeded in a 96-well plate at 1·10^5^ cells per well and cultured for 24 h in the presence or absence of the following TLR agonists: 100 ng/ml *S. minnesota* LPS (Sigma), 200 ng/ml recombinant flagellin (tlrl-flic, InvivoGen), 1 µg/ml Pam3CSK4 (tlrl-pms, InvivoGen), 100 µg/ml Zymosan A (Sigma), 1 µg/ml CL075 (tlrl-c75, InvivoGen) or 1 µg/ml CL087 (tlrl-c87, InvivoGen) in a final volume of 200 µl. Supernatants were taken and IL-23 (eBioscience), IL-1β (Biosource) IL-18 (MBL) and IL-12p70 (Sanquin) concentrations were determined by ELISA. For experiments with Mϕ1, cells were harvested with trypsin-EDTA, washed with PBS and seeded at 3.3·10^5^ per well in 24-well or 1·10^6^ in 12-well culture plates (Corning Life Sciences) and allowed to adhere. Subsequently Mϕ1 were stimulated with TLR agonists, infected with group B *Salmonella* at a 10∶1 multiplicity of infection as described below, or left unstimulated. Twenty-four hours after stimulation, supernatants were collected and cytokine production was determined by ELISA. To test the effect of GM-CSF and IFN-γ pre-stimulation, 1·10^5^ CD14^+^ monocytes were cultured for 16 hours with 50–5000 pg/ml GM-CSF, 50–5000 pg/ml IFN-γ (Biosource) or left unstimulated. Subsequently cells were stimulated with 100 ng/nl LPS plus 1 µg/ml CL075 for 24 hours, or left unstimulated. After stimulation cell free supernatants were collected and IL-23 production was determined by ELISA (eBioscience).

### Supernatant Transfer Experiments

To generate supernatants from *Salmonella* stimulated Mϕ1, 1·10^6^ cells were seeded in a 12-well plate and infected with group B *Salmonella* at a 10∶1 multiplicity of infection. To promote the uptake of bacteria, the bacteria were spun onto the macrophages by centrifugation at 300×*g* for 5 min and the cells were allowed to internalize the bacteria for 30 min at 37°C, 5% CO_2_. Cells were washed 3 times with PBS after which medium containing 50 µg/ml gentamicin (Sigma) was added to kill extracellular Salmonellae. After 30 minutes, medium was replaced with 2 ml fresh medium containing 10 µg/ml gentamicin. Twenty-four hours after infection, cell free supernatants were collected and used to stimulate anti-CD56 beads isolated cells. Overnight rested CD56^+^ cells were seeded 1·10^5^ cells per well in a 96-well plate and stimulated for 48 hr with 100 µl of conditioned Mϕ1 medium, with or without 100 ng/ml IL-18 (MBL), in a final volume of 200 µl. After 48 hours of culture, cell free supernatants were collected and IFN-γ production was measured by cytokine specific ELISA (Biosource). For cytokine neutralization assays, macrophage supernatants were pre-incubated with 10 µg/ml anti-IL-12p40 (C8.6, eBiosciences) or 10 µg/ml anti-IL-18 (MBL) or 5 µg/ml anti IL-1β (BD Biosciences) for 30 minutes at 37°C.

To generate supernatants of CD56^+^ cells, 1·10^6^ cells were stimulated with 10 ng/ml IL-23 plus 10 ng/ml IL-1β, or left unstimulated, for 24 hours. Cell free supernatants were collected and used to stimulate CD14^+^ monocytes. CD14^+^ cells were seeded 1·10^5^ cells per well in a 96-well plate and stimulated for 24 hr with 100 ng/ml LPS, in combination with 100 µl supernatant of CD56^+^ cells, 2.5 ng/ml IFN-γ (Biosource) or medium in a final volume of 200 µl. To neutralize IFN-γ, supernatants from stimulated CD56^+^ cells were pre-incubated with 2 µg/ml anti-IFN-γ (Genzyme) for 30 minutes at 37°C. Supernatants were collected and IL-12p70 was measured by cytokine specific ELISA (BD Bioscience).

### IFN-γ and GM-CSF Production

To determine GM-CSF and IFN-γ production in NK and NK-like T cells, overnight-rested CD56^+^ beads-isolated cells were seeded 1·10^5^ cells per well in a 96-well plate and stimulated for 24 hr with various concentrations of IL-23, alone or in combination with various concentrations of IL-18 (MBL) or IL-1β (Biosource) in a final volume of 200 µl. Concentrations of IFN-γ and GM-CSF in the cell free supernatants were determined by cytokine-specific ELISA (Biosource).

For intracellular staining of IFN-γ, CD56^+^ beads-isolated cells were seeded 10^5^ cells/well in 96-well plates and stimulated with IL-23 (R&D Systems), IL-18 (MBL), IL-1β (Biosource) or a combination of these cytokines for 48 hours. The last 5 hours of stimulation BD GolgiPlug (BD PharMingen) was added (final concentration 1∶1000). Cells were fixed in 4% paraformaldehyde (Sigma) and permeabilised in 90% methanol. Cells were stained with Alexa 647-labeled anti-human IFN-γ in combination with PE-labeled anti-human CD56 and FITC-labeled anti-human CD3 (BD PharMingen).

## Results

### Monocytes and Mϕ1 Produce IL-23, but Not IL-12, in Response to Various TLR Agonists

**Figure 1:**
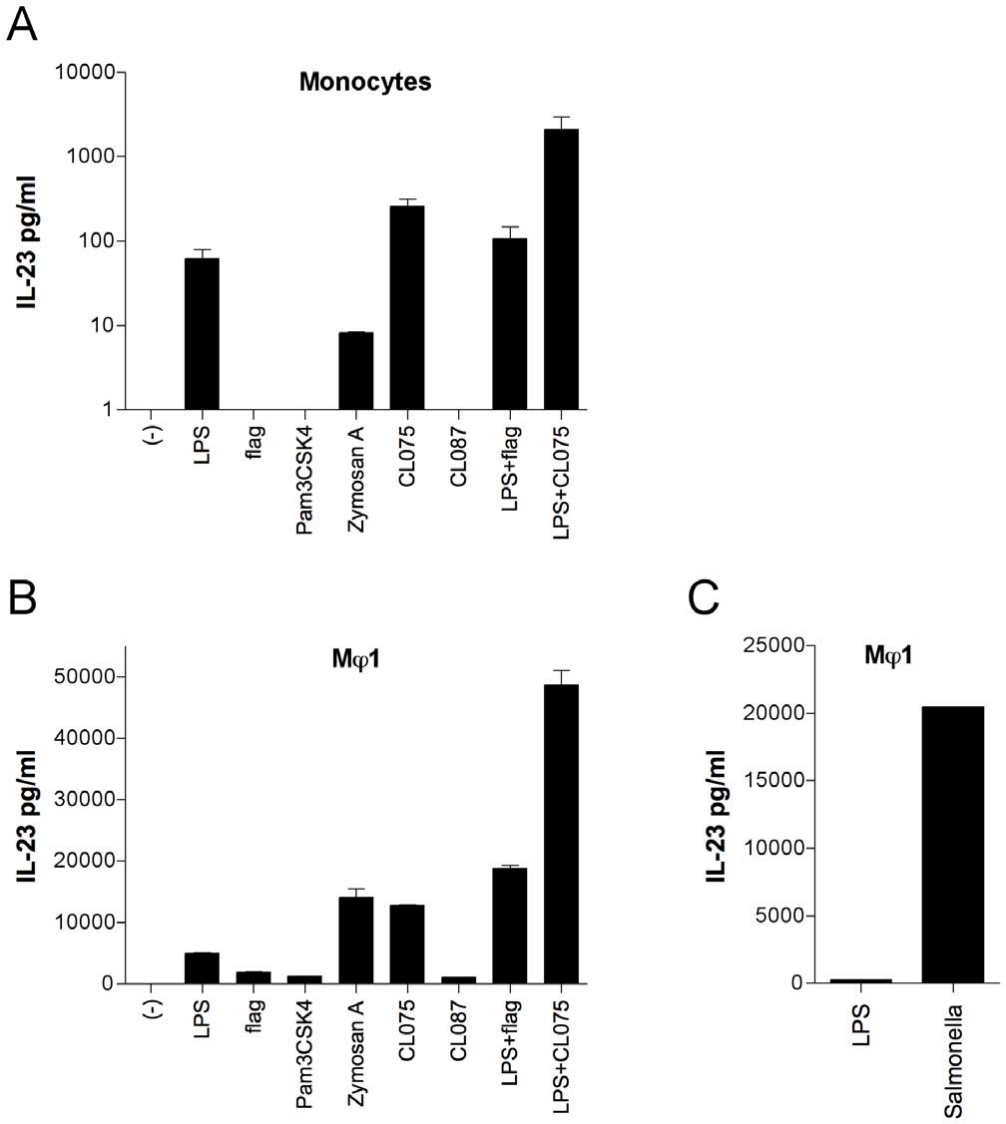
TLR stimuli and *Salmonella* infection induce IL-23 production in human monocytes and Mϕ1. 1 ·10^5^ overnight rested anti-CD14 beads-isolated monocytes (A) or 5 ·10^4^ Mϕ1 (B) were stimulated with 100 ng/ml LPS, 200 ng/ml flagellin (flag), 1 µg/ml Pam3CSK4, 100 µg/ml Zymosan A, 1 µg/ml CL075, 1 µg/ml CL087, 100 ng/ml LPS plus 200 ng/ml flagellin, LPS 100 ng/ml plus 1 µg/ml CL075 for 24 hours, or left unstimulated. (C) Mϕ1 were infected with live *Salmonella* with a multiplicity of infection (MOI) of 10, left uninfected or were stimulated with 100 ng/ml LPS. Supernatants were collected after 24 hours and IL-23 protein production was assessed by ELISA. Data are means±standard deviation (SD) of triplicates in one representative experiment of five (A and B) or means of duplicates in one representative experiment of three (C).

LPS is known to induce IL-23, but not IL-12, production in Mϕ1 [5]. To determine whether various TLR agonists are capable of inducing IL-23 or IL-12 production in monocytes and Mϕ1, we first tested these capacities in freshly isolated human monocytes. Unstimulated monocytes did not produce IL-23, whereas Zymosan A (agonist for TLR2/6) induced minimal IL-23 production, LPS (TLR4) and CL075 (TLR8/7) each induced small amounts of IL-23, while LPS and CL075 synergized in the induction of IL-23 production in monocytes (Fig. 1A). IL-23 production was also observed in response to LPS plus flagellin (TLR5) but the amount released was similar to that induced by LPS alone. Of note, the amounts of IL-23 produced by CD14^+^ monocytes varied somewhat between different donors, the relative amount released upon the various TLR stimulations was however similar (data not shown). In none of the supernatants IL-12p70 was detected, given a detection limit of the IL-12p70 ELISA of 3 pg/ml (data not shown).

Next, we compared the amount of IL-23 and IL-12 produced by monocytes to those by cultured Mϕ1. Upon incubation with each of the TLR agonists Mϕ1 produced IL-23 (Fig. 1B). LPS synergized with both CL075 and flagellin in the induction of IL-23. As compared with freshly isolated monocytes at identical cell number and incubation conditions, Mϕ1 produced markedly more IL-23 in response to the TLR agonists. Again, no IL-12p70 production was detected in response to any of the stimuli (data not shown). To verify that the Mφ1 used in these experiments are capable of producing IL-12p70 they were stimulated with LPS in combination with IFN-γ. Large amounts of IL-12p70 were detected in the supernatants (data not shown).

### Infection of Mϕ1 with Live Salmonella Induces IL-23, but Not IL-12, Production

To determine whether not only PAMPs such as the TLR agonists, but also live pathogens induce IL-23 rather than IL-12 production in Mϕ1, we infected cultured Mϕ1 with live *Salmonella* and assessed cytokine release in the culture supernatants. Mϕ1 exposed to and containing ingested *Salmonellae* produced IL-23 whereas Mϕ1 left unstimulated did not (Fig. 1C). Of note, *Salmonella* infected Mϕ1 produced markedly more IL-23 as compared with Mϕ1 stimulated with large amounts of LPS (Fig. 1C). Similar to stimulation with TLR agonists, we did not detect any IL-12p70 in the supernatants of infected Mϕ1 (data not shown). Moreover, in a related project, micro-array analysis of Mϕ1 infected with live *Salmonella* for 1, 2, 4, 8 and 24 hours revealed that infection did not induce *IL12A* (IL-12p35) transcription, whereas *IL12B* (IL-12p40) and *IL23A* (IL-23p19) transcription were both upregulated in response to *Salmonella* (van de Wetering, unpublished data).

### TLR Stimuli and Infection with Live Salmonella Induce IL-18 and IL-1β Production

**Figure 2:**
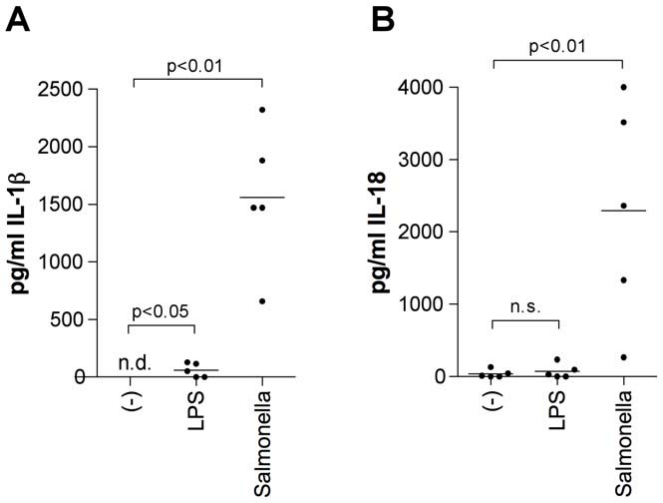
*Salmonella* infection induces IL-18 and IL-1β production in Mϕ1. 3.3 ·10^5^ Mϕ1 of five different donors were left unstimulated, stimulated with LPS or infected with *Salmonella* with a MOI of 10. Supernatants were collected after 24 hours and IL-18 (A) and IL-1β (B) production was measured by ELISA. Each dot represents one donor. The mean is indicated by a horizontal line. A paired two-tailed student's t-test was used for statistical analysis. n.d. = not detected.

We have shown previously that for IL-23 to induce IFN-γ production in NK-like T cells, an additional stimulus such as IL-18 is required [8]. Similar to IL-18, IL-1β can costimulate for IFN-γ production [16], [17]. To determine whether the production of IL-23 occurs in concert with that of IL-18 or IL-1β, we assayed the production of these cytokines by Mϕ1 in response to LPS or infection with *Salmonella*. Unstimulated cells produced minimal IL-18, but no IL-1β. Although IL-18 and IL-1β production in response to LPS and infection varied markedly between donors; Mϕ1 of all donors produced both IL-18 and IL-1β in response to these stimulations (Fig. 2A and B). *Salmonella*-infected Mϕ1 produced markedly more IL-18 and IL-1β as compared with cells stimulated with LPS (Fig. 2A and B).

### IL-23 and IL-1β Synergize in the Induction of IFN-γ in CD56^+^ Cells

**Figure 3:**
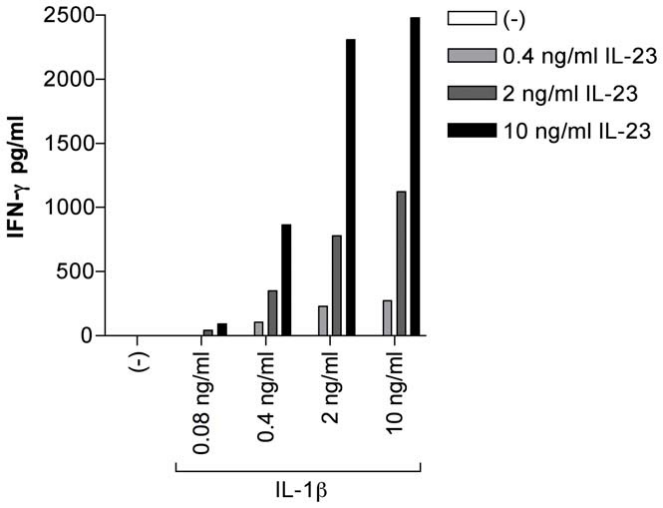
IL-23, in combination with IL-1β, induces IFN-γ production in primary human CD56^+^ cells. Anti-CD56 MACS bead isolated cells were rested overnight and then left unstimulated or stimulated with indicated concentrations of IL-23 in combination with various concentrations of IL-1β. Supernatants were collected 24 hours after stimulation and IFN-γ concentration was measured by ELISA. IL-23 and IL-1β synergize in the induction of IFN-γ. One representative experiment of three is shown.

**Figure 4:**
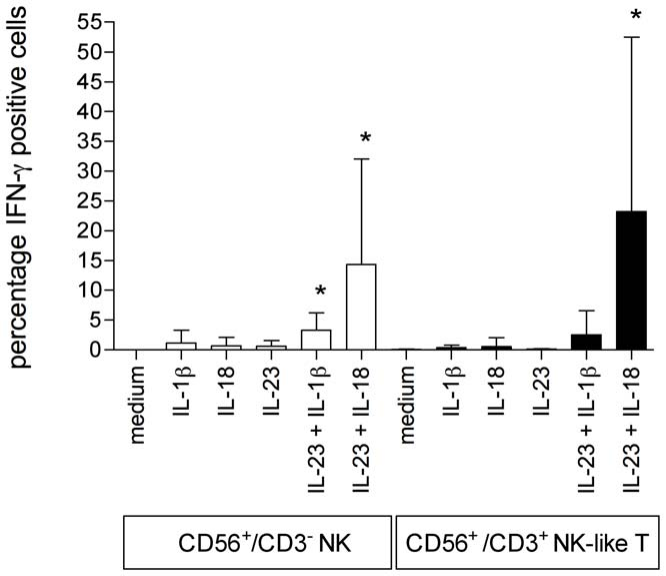
IL-23 in combination with IL-1β or IL-18 induces IFN-γ production in both CD56^+^/CD3^−^ NK cells and CD56^+^/CD3^+^ NK-like T cells. Anti-CD56 MACS bead isolated cells were rested overnight and then left unstimulated or stimulated with 10 ng/ml IL-1β, 100 ng/ml IL-18, 10 ng/ml IL-23, 10 ng/ml IL-1β plus 10 ng/ml IL-23 or 100 ng/ml IL-18 plus 10 ng/ml IL-23, for 48 hours. Cells were fixed, permeabilised and labeled with anti-human CD3-PE, anti-human CD56-FITC and anti-human IFN-γ-Alexa 647. Unstimulated cells and cells stimulated with a single cytokine do not produce significant amounts of IFN-γ. CD3^−^ NK cells stimulated with IL-1β or IL-18 in combination with IL-23 induce significant IFN-γ production, while in CD3^+^ NK-like Tcells only IL-23 plus IL-18 induces significant IFN-γ production. Data are means±SD of experiments with cells obtained from 8 donors. Two-tailed paired t-tests were performed of stimulated cells versus unstimulated cells. * indicates a p-value<0.05.

Infection of Mϕ1 with live Salmonella resulted in the production of IL-23, IL-18 and IL-1β. IL-23 is reported to induce IFN-γ production in NK, NK-like T cells and in T cells, in synergy with IL-18 [7], [8], [18]. As mentioned before, IL-1β is known to enhance IL-12 induced IFN-γ production [16], [17]. Therefore, we tested whether perhaps IL-23 in combination with IL-1β could also induce IFN-γ production in CD56^+^ (NK and NK-like T) cells. Each cytokine alone was not able to induce IFN-γ production (Fig. 3). However, when IL-23 and IL-1β were combined, they synergized in inducing IFN-γ production (Fig. 3). Isolated CD56^+^ cells consist of CD56^+^/CD3^−^ NK cells and CD56^+^/CD3^+^ NK-like T cells. We next determined which of these two populations were responsible for the observed IFN-γ production by IL-23 plus IL-1β stimulated CD56^+^ cells. Because sorting CD3^+^ cells with an anti-CD3 antibody induces undesired activation of the cells, we used intracellular staining for IFN-γ in combination with CD3/CD56 labeling. We arbitrarily designated a population positive for IFN-γ when more than 1% of the cells stained IFN-γ positive. CD56^+^ cells obtained from eight donors were stimulated with IL-23, IL-1β, IL-18, IL-23 plus IL-1β, IL-23 plus IL-18, or left unstimulated for 48 hours. No significant IFN-γ production was observed in unstimulated cells or cells stimulated with either IL-23, IL-1β or IL-18 (Fig. 4). Surprisingly, the cells producing IFN-γ in response to IL-23 plus IL-1β or IL-23 plus IL-18 varied between donors. In response to IL-23 plus IL-1β in half of the donors IFN-γ production was observed in both CD56^+^/CD3^−^ NK cells and CD56^+^/CD3^+^ NK-like T cells, whereas in the other half IFN-γ production was only observed in CD56^+^/CD3^−^ NK-like T cells (Table 1). In response to IL-23 plus IL-18 IFN-γ production was observed in both populations in seven donors, while in one donor only CD56^+^CD3^−^ cells produced IFN-γ (Table 1). On average, significant IFN-γ production was observed in response to IL-23 plus IL-1β or IL-18 in NK cells, while in NK-like T cells only IL-23 plus IL-1β resulted in significant IFN-γ production (Fig. 4).

**Table 1:** Percentages of IFN-γ producing CD56^+^ cells after stimulation with cytokines.

		Stimulation					
		-	IL-23
Donor	Fraction	-	IL-1β	IL-18	-	IL-1β	IL-18
1	CD3^−^	0,2	0,8	0,2	0,4	3,0	6,7
	CD3^+^	0,0	0,1	0,0	0,1	0,2	0,7
2	CD3^−^	0,1	0,3	0,2	0,3	1,9	4,4
	CD3^+^	0,1	0,1	0,1	0,2	0,6	4,8
3	CD3^−^	0,1	0,1	0,0	0,2	1,5	7,5
	CD3^+^	0,1	1,2	0,0	0,1	11,6	35,9
4	CD3^−^	0,1	0,3	0,0	0,1	3,2	53,4
	CD3^+^	0,0	0,4	0,0	0,0	1,2	84,1
5	CD3^−^	0,2	6,1	3,9	2,8	9,7	15,8
	CD3^+^	0,1	0,6	3,9	0,3	2,3	19,5
6	CD3^−^	0,0	0,1	0,1	0,1	1,2	3,4
	CD3^+^	0,1	0,0	0,0	0,0	0,5	10,0
7	CD3^−^	0,1	0,1	0,2	0,5	2,4	9,0
	CD3^+^	0,1	0,2	0,0	0,1	1,0	7,5
8	CD3^−^	0,1	0,3	0,0	1,0	4,7	17,8
	CD3^+^	0,0	0,0	0,0	0,0	0,1	2,7

Percentages of IFN-γ producing CD56^+^/CD3^−^ NK cells and CD56^+^/CD3^+^ NK-like T cells for each of eight donors after stimulation of CD56^+^ cells with the indicated cytokines for 48 h.

Since IL-23 in combination with IL-1β is known to promote the production of IL-17 in memory T cells [19], we also analyzed IL-17 production by CD56^+^ cells in response to IL-23 and IL-1β. However, in none of the supernatants IL-17 was detected (data not shown).

### Supernatants of Salmonella Infected Mϕ1 Induce IFN-γ Production in Primary CD56^+^ Cells

**Figure 5:**
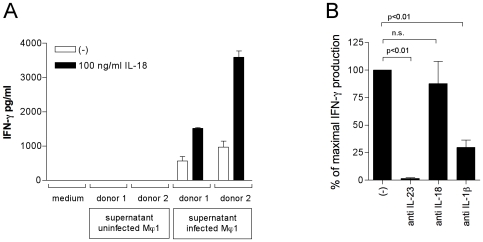
Supernatants of *Salmonella*-infected Mϕ1 induce IFN-γ production in primary human CD56^+^ cells. (A) IFN-γ production by CD56^+^ NK/NK-like T cells from two donors. Cells were cultured for 48 hours in the presence of supernatants obtained from uninfected or *Salmonella* infected Mϕ1 (from two donors) with or without additional exogenous recombinant IL-18. As a control, medium only was used. Data are means±SD of triplicates in one representative experiment of three. (B) IFN-γ production in CD56^+^ cells induced by supernatants of *Salmonella*-infected Mϕ1 is blocked by neutralizing IL-12p40 or IL-1β, but not by neutralizing IL-18. CD56^+^ NK/NK-like T cells were cultured for 48 hours in the presence or absence of supernatant obtained from Mϕ1 cells infected with *Salmonella*, in the presence or absence of an IL-12p40, IL-18 or IL-1β neutralizing antibody. IFN-γ concentration was determined by ELISA. IFN-γ production induced by supernatants of *Salmonella* infected Mϕ1 is set at 100%. A paired two-tailed student's t-test was used for statistical analysis. Data are means±SEM of experiments with cells obtained from 10 different donors.

So far we found that IL-23, IL-18 and IL-1β, but not IL-12p70, are present in supernatants of *Salmonella* infected Mϕ1. We showed that IL-23 can induce IFN-γ production in CD56^+^ when IL-1β is present. Previously, we have shown that CD56^+^ cells produce IFN-γ when stimulated with IL-23 plus IL-18, in the absence of IL-12 [8]. Based on these data we expected that supernatants from *Salmonella* infected Mϕ1 would be able to induce IFN-γ production in CD56^+^ cells. Supernatants of *Salmonella* infected Mϕ1 were indeed able to induce IFN-γ production in primary CD56^+^ cells (Fig. 5A). Neutralization of LPS in these supernatants did not alter their ability to induce IFN-γ production (data not shown).

Since IL-18 is induced in small amounts only, even after infection by *Salmonella*, we determined whether IL-18 was a limiting factor in the induction of IFN-γ in CD56^+^ cells by supernatants of Mϕ1 infected with *Salmonella*. The addition of recombinant IL-18 to the supernatants increased the IFN-γ production by these CD56^+^ cells (Fig. 5A).

### IL-23 and IL-1β, but Not IL-18, in Supernatants Are Critical for the Induction of IFN-γ Production in CD56^+^ Cells

To verify the role of IL-23 in the induction of IFN-γ by supernatants of *Salmonella* infected Mϕ1, we neutralized IL-23 using an antibody binding the p40 subunit of IL-23. (Note that although the p40 antibody can also neutralize IL-12, we have shown above that no IL-12 was present in these supernatants). When IL-23 was thus neutralized, IFN-γ production was effectively abrogated (Fig. 5B), even when exogenous IL-18 was added (data not shown).

Similarly, to specify the role of IL-18 and IL-1β in the supernatants of *Salmonella*-infected Mϕ1 in the induction of IFN-γ production, we used a specific antibody to neutralize these interleukins. Pilot experiments had confirmed that the IL-18 antibody could neutralize a large amount of recombinant IL-18 (4 ng/ml) effectively (data not shown). Although exogenous IL-18 enhanced the Mϕ1 supernatant-induced IFN-γ production by CD56^+^ cells, neutralization of IL-18 in this supernatant did not affect the production of IFN-γ significantly (Fig. 5B), suggesting IL-18 is not crucial in this respect. In contrast, neutralization of IL-1β reduced the capacity of the supernatants to induce IFN-γ production significantly (Fig. 5B).

### IL-23, in Synergy with IL-18 or IL-1β, Induces GM-CSF Production in CD56^+^ NK/NK-Like T Cells

**Figure 6:**
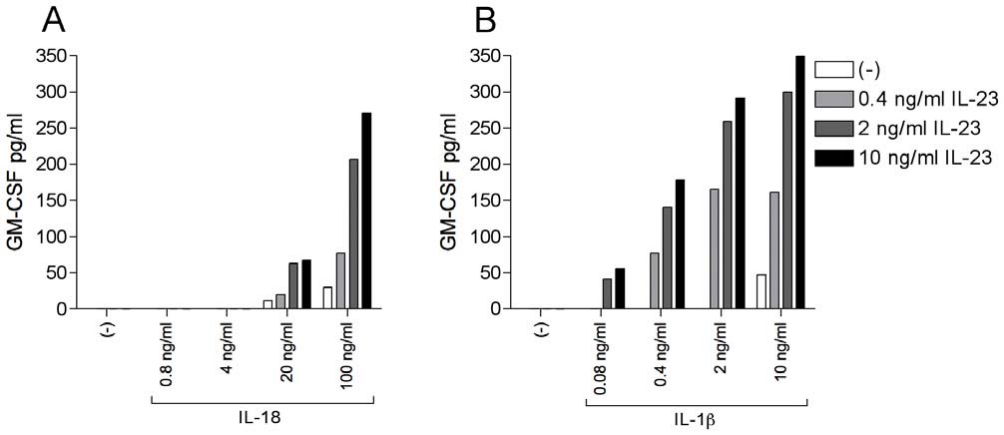
IL-23 in synergy with IL-18 or IL-1β induces GM-CSF in CD56^+^ cells. 1·10^5^ anti-CD56 beads isolated cells were rested for 24 hours and then left unstimulated or stimulated with indicated concentrations of IL-23 in combination with various concentrations of IL-18 (A) or IL-1β (B). IL-18 or a combination of these cytokines. Supernatants were collected 24 hours after stimulation and GM-CSF protein production was measured by ELISA. One representative experiment of three is shown.

IL-18 is known to induce GM-CSF production in NK cells in combination with various cytokines [10]. To determine whether IL-18 or IL-1β, combined with IL-23 can induce GM-CSF production in human CD56^+^ NK/NK-like T cells, these cells were stimulated with IL-18, IL-1β or IL-23 and combinations of these cytokines, or left unstimulated for 24 hours. Unstimulated cells and cells stimulated with IL-23 alone did not produce any GM-CSF (Fig. 6A). In contrast, IL-18 alone induced a small amount of GM-CSF, when high concentrations were used. In combination with IL-23 on the other hand, IL-18 induced considerable amounts of GM-CSF in CD56^+^ cells (Fig. 6A). CD56^+^ cells stimulated with high concentrations of IL-1β produced GM-CSF, whereas cells stimulated with lower concentrations of IL-1β did not. IL-23 synergized with IL-1β in inducing GM-CSF (Fig. 6B). For both IL-18 and IL-1β, strongest synergy was observed at highest concentrations.

### GM-CSF and IFN-γ Prime Monocytes for Enhanced IL-23 Production

**Figure 7:**
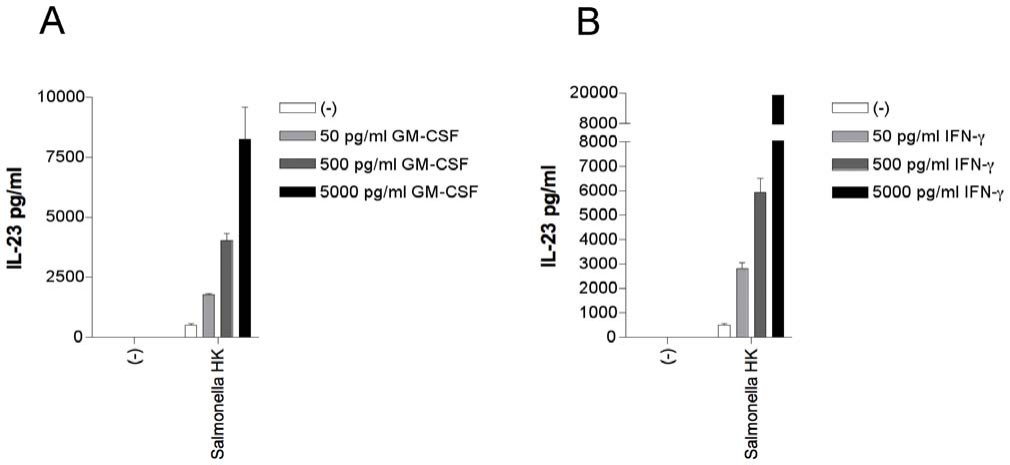
GM-CSF and IFN-γ prime monocytes for enhanced IL-23 production. 1 ·10^5^ CD14^+^ monocytes were prestimulated with GM-CSF (A) or IFN-γ (B) for 16 hours in concentrations indicated. Subsequently cells were stimulated with heat killed Salmonella (moi = 10) or left unstimulated for 24 hours. Supernatants were taken and IL-23 production was measured by ELISA. Data are means±SD of triplicates in one representative experiment of five.

Mϕ1 generated by culturing CD14^+^ monocytes for six days in the presence of GM-CSF are strong producers of IL-23 compared with freshly isolated CD14^+^ monocytes ([5] and above). To investigate whether GM-CSF or IFN-γ (known to enhance the expression of the IL-23 subunit IL-12p40 [20]) could prime monocytes directly for enhanced IL-23 production in response to stimulation with heat killed *Salmonella*, we pre-stimulated CD14^+^ cells for 16 hours with GM-CSF or IFN-γ and assessed IL-23 production. Stimulation of monocytes with GM-CSF or IFN-γ alone did not induce IL-23 production (Fig. 7A and 7B). However, sixteen hours of pre-stimulation of monocytes with as little as 50 pg/ml GM-CSF enhanced IL-23 production in response to heat killed *Salmonella* (Fig. 7A), with a dose dependent priming effect (Fig. 7A). Similar to GM-CSF, pre-stimulation of monocytes with IFN-γ also enhanced IL-23 production in response to heat killed *Salmonella*, again in a dose dependent manner (Fig. 7B).

### IL-23 Plus IL-1β Stimulated CD56^+^ Cells Prime Monocytes for IL-12p70 Production

**Figure 8:**
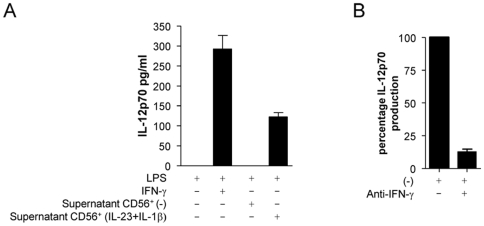
IFN-γ in supernatants of IL-23 plus IL-1β stimulated CD56^+^ cells primes monocytes for IL-12p70 production in response to LPS. 1 ·10^5^ CD14^+^ monocytes were stimulated with 100 ng/ml LPS in combination with 2.5 ng/ml IFN-γ, supernatants of unstimulated CD56^+^ cells, supernatants of IL-23 plus IL-1β stimulated CD56^+^ cells, or medium alone for 24 hours (A). 1 ·10^5^ CD14^+^ monocytes were stimulated with 100 ng/ml LPS in combination supernatants of IL-23 plus IL-1β stimulated CD56+ cells, plus or minus 2 µg/ml anti-IFN-γ for 24 hours (B). Supernatants were taken and IL-12p70 production was measured by ELISA. Data are means±SD of triplicates from one representative experiment of three.

To produce IL-12p70 in response to LPS, IFN-γ is needed as an extra stimulus. CD56^+^ cells produce IFN-γ in response to IL-23 plus IL-1β, therefore we wanted to know whether supernatants of IL-23/IL-1β stimulated CD56^+^ cells could prime monocytes for IL-12p70 production in response to LPS. Monocytes were stimulated with LPS, plus or minus supernatants of IL23/IL-1β stimulated CD56^+^ cells or, as a positive control, IFN-γ. Monocytes stimulated with LPS alone did not produce IL-12p70, whereas cells stimulated with LPS in combination with IFN-γ or supernatants of IL-23 plus IL-1β stimulated CD56^+^ cells both produced IL-12p70 (Fig. 8A). To assess the role of IFN-γ present in the supernatants of IL-23 plus IL-1β stimulated CD56^+^ cells in the priming effect on IL-12p70 production by monocytes, we used an IFN-γ neutralizing antibody. Neutralization of IFN-γ greatly diminished the priming effect of these supernatants on LPS induced IL-12p70 production by monocytes (Fig. 8B). Of note, IL-23 plus IL-1β did not directly prime monocytes to produce IL-12p70 in response to LPS (data not shown).

## Discussion

In this study we demonstrate that human monocytes and cultured pro-inflammatory macrophages (i.e., Mϕ1) both produce IL-23, IL-18 and IL-1β in response to various TLR agonists, during co-incubation and upon ingestion of live *Salmonella*. Subsequently, in response to IL-23 in combination with IL-18 or IL-1β, CD56^+^ cells produce IFN-γ and GM-CSF. These cytokines in turn enhance IL-23 production by monocytes and macrophages in response to TLR agonists, implying a positive feedback loop. Furthermore, supernatants of *Salmonella* infected Mϕ1 induce IFN-γ production in CD56^+^ cells and this induction of IFN-γ was critically dependent on IL-23 and IL-1β. Finally, supernatants from IL-23 plus IL-1β stimulated CD56^+^ cells allowed monocytes to produce IL-12p70 in response to LPS.

The assumption of an IL-23 positive feedback loop is based on the following observations: (**1**) We demonstrated that monocytes as well as Mϕ1 are able to produce IL-23 in response to various TLR agonists. In addition, monocytes and Mϕ1 produced small amounts of IL-18 and IL-1β in response to individual TLR agonists, while cells infected with live *Salmonella* produced large amounts of IL-18, IL-1β and IL-23. In line, Verreck *et al.* showed that Mϕ1 produce IL-23 and IL-1β in response to LPS or mycobacterial sonicate [21]. (**2**) Guia *et al.* reported that in the presence of macrophages infected with *Salmonella* in vitro, CD56^+^ NK-like T cells, but not CD56^−^ T cells produce IFN-γ in absence of TCR stimulation [22]. Here we show that IL-23 and IL-1β in these supernatants of *Salmonella* infected Mϕ1 were critical components for the induction of IFN-γ production in the CD56^+^ NK/NK-like T cells, because neutralization of these interleukins resulted in abrogation or a strong reduction of IFN-γ production, respectively. (**3**) Recombinant IL-18 was able to enhance the induction of IFN-γ in CD56^+^ in response to supernatants of *Salmonella* infected Mϕ1. However, neutralization of IL-18 did not significantly reduce the IFN-γ production. These results support that IL-23 and IL-1β, but not IL-18, in the supernatant of *Salmonella* infected Mϕ1 are critical for the induction of IFN-γ in CD56^+^ cells. Moreover, in CD56^+^ cells stimulated with IL-23 no synergy is seen with IL-18 when concentrations lower than 20 ng/ml IL-18 are used [8], indicating that the IL-18 concentration induced by *Salmonella* (which was ∼1–4 ng/ml) may be too low to synergize with IL-23 in inducing IFN-γ. In addition, *Salmonella* infection is known to induce TNF production in macrophages and TNF has been reported to enhance IFN-γ production [17], [23]. Therefore, in our experiments, TNF in the supernatants of infected Mϕ1 may also enhance IFN-γ production. However, we did not determine the role of TNF in this respect. (**4**) IFN-γ and GM-CSF both enhanced IL-23 production in monocytes in response to various stimulations. (**5**) Moreover, both cytokines could be induced by IL-23, in combination with IL-18 or IL-1β, in CD56^+^ cells. Depending on the concentrations used, IL-23 plus IL-18 or IL-1β induced more than 200 pg/ml GM-CSF, a concentration which we have shown to be sufficient to prime CD14^+^ monocytes for enhanced IL-23 production.

Together these observations confirm the presence of a positive feedback loop in which IL-23 can enhance its own expression via the induction of IFN-γ and GM-CSF. In line with our observations, IFN-γ was recently reported to enhance *Francisella tularensis* induced IL-23 in human monocytes [24]. IFN-γ allows for the transcription of IL-12p35 in response to LPS [25] and for the induction of IL-12 in response to a PAMP an additional stimulus such as IFN-γ is needed [5]. Herein we show that IL-23 together with IL-1β present in supernatants of *Salmonella* infected Mϕ1 can provide this costimulus by the induction of IFN-γ in NK-like T cells, thereby allowing subsequent IL-12p70 production in response to *Salmonella* and the initiation of a Th1 response. In contrast, in human DCs IFN-γ can inhibit IL-23p19 mRNA transcription [26], emphasizing the complexity of the regulation of this cytokine.

In this study we report on a positive feedback of IL-23 production, but negative feedback mechanisms may exist as well. For instance, IL-4 and IL-10 are both reported to inhibit the induction of IL-23 and may serve as a negative feedback [27]. IL-4 may be important in balancing IL-23 and IL-12 production as IL-4 can enhance IL-12p70 production [28]–[30]. It would be interesting to explore the effects of these and other potential negative regulators of IL-23.

To reach the conclusion on a positive feedback loop in which IL-23 enhances its own expression via the induction of IFN-γ and GM-CSF, the following pitfalls of this study need to be considered. Firstly, in supernatants of Mϕ1 infected with live *Salmonella*, we neutralized IL-23 using an antibody which is able to neutralize IL-12p70 as well. Though we did not detect IL-12p70 and IL-12p35 mRNA expression was not induced by infection with *Salmonella*, one should bear in mind that the detection limit of the IL-12p70 ELISA used was 3 pg/ml and that we can not exclude that the effect observed after neutralizing the IL-23 is due to the neutralization of small, undetectable amounts of IL-12p70. Secondly, the concentrations of the TLR agonists used may exceed physiological relevant conditions. For example, the concentrations of *Salmonella* LPS added to the cells used in this study are likely to differ from LPS concentration when Mϕ1 are being infected with live *Salmonella*. During sepsis 5–10 pg/ml LPS can be detected in the blood [31]. Thirdly, between donors we observed interindividual differences in cytokine production in response to the stimulations used. This may reflect differences in responsiveness to stimulation and differences in capacity to produce cytokines between cells obtained from different donors. We observed for example remarkable interindividual differences in the subset of IFN-γ producing CD56^+^ cells in response to IL-23 in combination with IL-1β or IL-18.

In addition to the elucidation of the positive feed-back loop of IL-23 expression we observed a synergy between LPS (TLR4) and flagellin (TLR5) and between LPS and CL075 (TLR8/7) with respect to the induction of IL-23 production in monocytes and Mϕ1. During an infection, synergy between TLRs is likely of importance because pathogens express multiple TLR agonists. LPS and flagellin for instance, which in synergy induce large quantities of IL-23 in Mϕ1, are both expressed by *Salmonella*. Synergy between LPS (TLR4) and R848, another TLR8/7 agonist, has been observed in the induction of IL-12 and IL-23 in human DCs [32]. Both TLR8 and TLR4 are implicated in the resistance against *M. tuberculosis* [33], [34], suggesting the synergy we observed between the TLR4 and the TLR8/7 agonists may be relevant in mycobacterial infections.

In conclusion, we have shown that various TLR agonists and infection with *Salmonella* can induce IL-23, IL-18 and IL-1β, but not IL-12, production in monocytes and Mϕ1. Furthermore, our findings indicate that a positive feedback loop exists in which IL-23 can enhance its own production via the induction of IFN-γ and GM-CSF, which both prime monocytes for enhanced IL-23 production. Last, IL-23, in combination with IL-1β, could prime monocytes to produce IL-12p70 in response to LPS, via the activation of CD56^+^ cells, thereby amplifying the type-1 cytokine pathway to IFN-γ production.

IL-23 plus IL1β induced IFN-γ production in CD56^+^ cells, which consist of CD56^+^/CD3^−^ NK cells and CD56^+^CD3^+^ NK-like T cells. In half of the donors IFN-γ production was observed in both NK cells and NK-like T cells, whereas in the other half IFN-γ production was observed only in the NK cells. Possible explanations for these interindividual differences may be previous (recent) exposure to different pathogens or genetic differences between donors; these may be elucidated when individual donors are assayed at regular intervals over a longer time period to determine whether or not the individual cytokine production profiles are constant.
